# Antibacterial Mechanisms of Zinc Oxide Nanoparticle against Bacterial Food Pathogens Resistant to Beta-Lactam Antibiotics

**DOI:** 10.3390/molecules27082489

**Published:** 2022-04-12

**Authors:** Rajapandiyan Krishnamoorthy, Jegan Athinarayanan, Vaiyapuri Subbarayan Periyasamy, Mohammad A. Alshuniaber, Ghedeir Alshammari, Mohammed Jamal Hakeem, Mohammed Asif Ahmed, Ali A. Alshatwi

**Affiliations:** 1 Nanobiotechnology and Molecular Biology Research Lab, Department of Food Science and Nutrition, College of Food and Agriculture Sciences, King Saud University, Riyadh 11541, Saudi Arabia or jegan.dna@gmail.com (J.A.); or vsperrys@gmail.com (V.S.P.); malshuniaber@ksu.edu.sa (M.A.A.); 2 Department of Food and Nutrition, College of Food and Agriculture Sciences, King Saud University, Riyadh 11541, Saudi Arabia; aghedeir@ksu.edu.sa (G.A.); mhakeem@ksu.edu.sa (M.J.H.); masifa@ksu.edu.sa (M.A.A.)

**Keywords:** food pathogens, beta-lactamase, membrane disintegration, broad spectrum, nanoparticles

## Abstract

The increase in β-lactam-resistant Gram-negative bacteria is a severe recurrent problem in the food industry for both producers and consumers. The development of nanotechnology and nanomaterial applications has transformed many features in food science. The antibacterial activity of zinc oxide nanoparticles (ZnO NPs) and their mechanism of action on β-lactam-resistant Gram-negative food pathogens, such as *Escherichia coli*, *Pseudomonas aeruginosa*, *Salmonella typhi*, *Serratia marcescens*, *Klebsiella pneumoniae*, and *Proteus mirabilis*, are investigated in the present paper. The study results demonstrate that ZnO NPs possesses broad-spectrum action against these β-lactamase-producing strains. The minimal inhibitory and minimal bactericidal concentrations vary from 0.04 to 0.08 and 0.12 to 0.24 mg/mL, respectively. The ZnO NPs elevate the level of reactive oxygen species (ROS) and malondialdehyde in the bacterial cells as membrane lipid peroxidation. It has been confirmed from the transmission electron microscopy image of the treated bacterial cells that ZnO NPs diminish the permeable membrane, denature the intracellular proteins, cause DNA damage, and cause membrane leakage. Based on these findings, the action of ZnO NPs has been attributed to the fact that broad-spectrum antibacterial action against β-lactam-resistant Gram-negative food pathogens is mediated by Zn^2+^ ion-induced oxidative stress, actions via lipid peroxidation and membrane damage, subsequently resulting in depletion, leading to β-lactamase enzyme inhibition, intracellular protein inactivation, DNA damage, and eventually cell death. Based on the findings of the present study, ZnO NPs can be recommended as potent broad-spectrum antibacterial agents against β-lactam-resistant Gram-negative pathogenic strains.

## 1. Introduction

Foodborne pathogens are significant causative agents of foodborne disease and malady, therefore causing a severe threat to food safety. The World Health Organization reports that 2 billion people are affected annually by foodborne diseases in developed and developing countries [1], and produce significant morbidity and mortality rates [2]. Some foodborne pathogens include *Escherichia coli*, *Pseudomonas aeruginosa*, *Salmonella typhi*, *Serratia marcescens*, *Listeria monocytogenes*, and *Klebsiella pneumoniae*, and *Proteus mirabilis* is the most important pathogen in the food industry [3,4,5]. A recent study estimated that the growing demand for food production in middle- and low-income countries will lead to 69% of global antibiotic use between 2010 and 2030 [6]. In recent years, foodborne outbreaks of pathogens have been associated with antimicrobial resistance [7]. Antimicrobial resistance in food pathogens is linked to antibiotic use in food animals, aquacultures, other agriculture products, soil and water, and even food preservation [8]. It has been reported that food animals, food products, and the food processing industry can be the primary reservoirs of multiple drug-resistant (MDR) bacteria. Such a scenario may play a role in disseminating drug-resistant bacteria to humans via food [9]. On the other hand, a food industry environment rich in nutrients confers many advantages to the pathogens that can easily bind to surfaces. Additionally, the food equipment’s surfaces, such as milk tanks, cheese tanks, butter centrifuges, pasteurizers, rubbers, polyethylene, polypropylene, and packing materials, are influenced by the surface properties, such as roughness, and hydrophobic interactions. These properties augment bacterial interfaces to surface adhesion by extracellular organelles, such as extracellular polymeric substances, flagella, and pili, during food processing or production. In this stage, environmental factors influence the interactions between the bacteria and surface, resulting in an irreversible adhesion, and regulate the signals to facilitate the formation of the MDR bacterial colony. Additionally, MDR bacterial colonization is more stable against physical, mechanical, and chemical processes, such as desiccation, liquid stream in the pipeline, chemicals, and disinfectant, used in the food industry [10]. Hence, critical hindrances to the food processing industry, including poultry and meat, dairy, and seafood [11], are prevalent. 

The survival of drug-resistant (DR) bacteria, especially β-lactam-resistant bacteria, has become an increasingly relevant issue in the food sector due to extensive applications in food processing, from production to consumption [12]. Beta-lactam and the fluoroquinolone class of antimicrobials are the primary therapeutic choices in the agriculture, food, veterinary, and health sectors [13]. Beta-lactam antibiotics consist of a 4-membered beta-lactam ring targeting the substrates of tanspeptidase and carboxypeptidase that mediate cell wall biosynthesis [14]. Beta-lactam antibiotics are one of the most prescribed antibiotics (involved in 60% of antibiotics); thus, the frequent and quantitative use collectively promotes the survival of bacteria via the development of a resistance against these antimicrobials [15]. The β-lactam-resistant bacterial strains hydrolyze the penicillins, cephalosporins, monobactams, and carbapenems by producing a β-lactamase enzyme, thereby inactivating these drugs and remaining resistant. Currently, different types of β-lactamase have been reported, including penicillinases, extended-spectrum β-lactamases (ESBLs), cephalosporinases (AmpCs), metallo lactamases (MBLs), and carbapenemases (KPCs) [16]. Gram-negative bacteria are the predominant group of pathogens in the food and agriculture industry and β-lactamase producers [17,18]. Currently, β-lactam-resistant bacterial strains are categorized as number three out of the top six dangerous bacterial pathogens listed by the Infectious Diseases Society of America [19]. Studies reported the progression of β-lactamase producing bacterial pathogens in livestock, raw materials, food processing equipment, and food animals [20,21,22]. The first, second, and third-generation cephalosporin groups of antibiotics, penicillin, and aztreonam, are the outdated antimicrobials for these strains [23]. Moreover, β-lactamase strains have altered outer-membrane proteins, penicillin-binding proteins, efflux pumps, and enhanced metabolisms [24]. Additionally, these strains can transfer resistance genes through mobile genetic elements, insertion sequences, and integrons; hence, previous studies showed an increased incidence of β-lactam resistance in raw vegetables, food animals, food products, food packing materials, and ready-to-eat foods [25]. Hence, the indulgence of nanomaterial applications has gained much more attention, due to their exciting properties, making them competent for food processing [26,27]. Among these, zinc oxide nanoparticles (ZnO NPs) attracted more attention due to their multifaceted properties, such as stability, a high surface-to-volume ratio, chemical reactivity, electrical and magnetic properties, non-cytotoxicity, biosafety, and biocompatibility, and are barely prone to bacterial resistance [28]. Some studies proposed that antibacterial activity and bactericidal actions occur via the generation of hydrogen peroxide [29,30]; other studies suggest that the binding action of ZnO NPs to the bacterial surface causes an electrostatic force, resulting in membrane damage and cell death [31]. However, the antibacterial potential of ZnO NPs and their mechanisms of action have not been studied on β-lactamase strains. Hence, in the present study, we aim to synthesize ZnO NPs using zinc oxide, and investigate their antibacterial activity against β-lactam-resistant strains. The outcome of ours study will help to discover suitable alternatives for food-packing applications. 

## 2. Results

### 2.1. The Characterization of ZnO NPs

**Figure 1:**
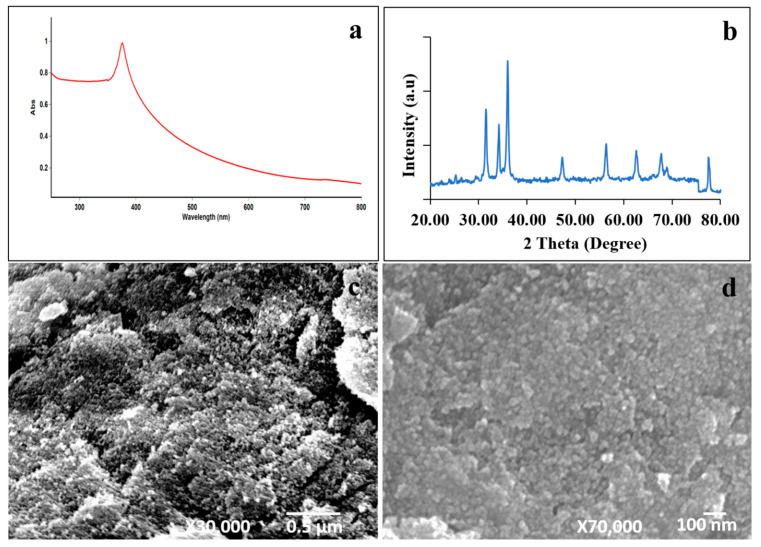
The size and morphology analysis of (**a**) UV-Vis absorption spectrum, (**b**) X-ray diffraction spectra, and (**c**,**d**) scanning electron microscopic images of the synthesized ZnO NPs.

**Figure 2:**
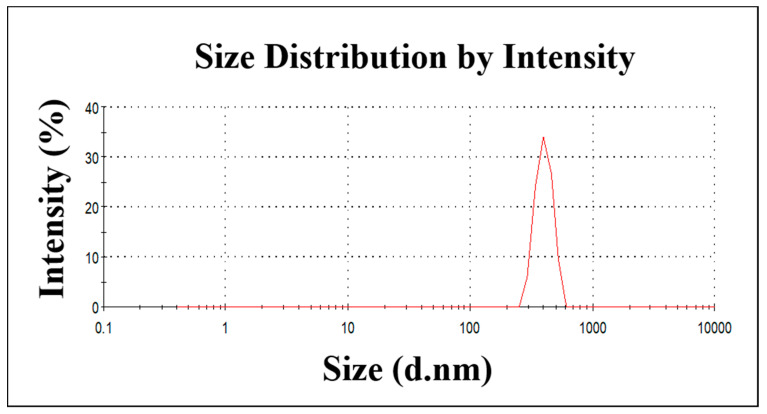
The particle-size distribution of the synthesized ZnO NPs.

The ZnO NPs were synthesized using the microwave-assisted method. The absorption spectrum of synthesized ZnO NPs in the UV-Vis spectra is shown in Figure 1a. We observed the sharp absorption maximum at a wavelength of 375 nm in synthesized ZnO NPs, which is attributed to the ZnO π–π* electronic excitation [32]. Figure 1b shows the XRD pattern of the prepared ZnO NPs. The ZnO NPs show sharp diffraction peaks located at 2θ = 31.6°, 34.2°, 36°, 47.3°, 56.4°, 62.6°, and 67.8°, which corresponds to the (100), (002), (101), (102), (110), (103), and (112) planes, respectively. There is no other diffraction peak, which confirms the presence of synthesized ZnO NPs without other impurities. These results indicate a hexagonal wurtzite-type ZnO NP formation. Additionally, the XRD result matches the JCPDS data 36-1451. The morphology of the synthesized ZnO NPs was analyzed by SEM micrographs. The obtained images are depicted in Figure 1c,d. The image clearly shows that ZnO NPs have a spherical shape, with an average particle size ranging from 60–80 nm. Our results suggest that microwave irradiation triggers ZnO NP formations. Additionally, we assessed the ZnO NPs’ particle-size distribution using dynamic light scattering. Figure 2 shows that the average particle size of ZnO NPs is 456 nm. These results suggest that ZnO NPs are aggregated in water. Overall, the physicochemical analysis results indicate ZnO NP formations. 

### 2.2. The In Vitro Antibacterial Activity of ZnO NPs against β-Lactam-Resistant Bacterial Strains

The ZnO NPs demonstrated robust anti-microbial activity against all selected β-lactam-resistant bacterial food pathogens. The MIC and MBC of the ZnO NPs are shown in Table 1. 

The MIC and MBC concentrations varied from 0.04 to 0.08 and 0.12 to 0.24 mg/mL, respectively. Non-β-lactamase-producing bacterial strains were the most susceptible organisms, with an MIC of 0.04 and MBC of 0.12 mg/mL. Among the β-lactam-resistant strains, *K. pneumoniae* (ATCC) *E. coli*, *P. aeruginosa*, *S. marcescens*, and *K. pneumoniae* exhibited MIC at 0.04 mg/mL, except for *P. mirabilis* and *S. typhi* (MIC-0.08 mg/mL). The variations were observed at MBCs between the β-lactam-resistant strains of *K. pneumoniae* (ATCC) *E. coli*, *S. marcescens* (0.2 mg/mL), and *S. typhi*, *P. aeruginosa*, *K. pneumoniae*, and *P. mirabilis* (0.24 mg/mL). 

### 2.3. The Inhibition of β-Lactamase Activity 

**Figure 3:**
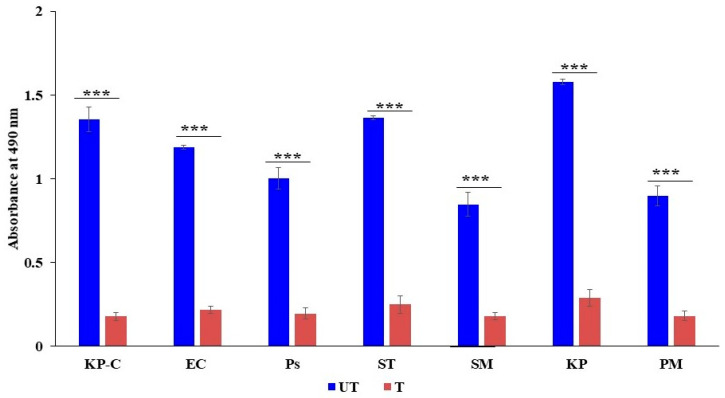
The dynamics of ZnO NPs on beta-lactamase activity. UT: untreated control; T: ZnO NPS treated; KP C: *K. pneumoniae* ATCC 700603; EC: *E. coli*; PS: *P. aeruginosa*; ST: *S. typhi*; SM: *S. marcescens*; KP: *K. pneumoniae*; and PM: *P. mirabilis.* The data are the averages of the triplicates and the error bar represents the mean ± SD. Compared to the ZnO NP free control *** *p* < 0.001.

Nitrocefin is a chromogenic cephalosporin, and β-lactamase hydrolyzes the nitrocefin results in the generation of a colored product, which can be detectable at 490 nm. The formation of colored products is directly proportional to β-lactamase activity. This study found that colored-product formation was high in all untreated bacterial pathogens; hence, the high OD value was recorded. However, the lowest OD values were found on all ZnO NP-treated cells, which indicates the least hydrolysis due to less β-lactamase activity. The present results suggest that ZnO NPs can be potent β-lactamase inhibitors (Figure 3). Interestingly, our study results found variations in β-lactamase activity among the untreated strains. The highest activity was exhibited in food pathogenic *K. pneumoniae*, *S. typhi*, *K. pneumoniae* control strain, *E. coli*, and *P. aeruginosa*, while *P. mirabilis* and *S. marcescens* showed low activity compared to the other tested strains. All the obtained values are statistically significant. 

### 2.4. The Generation of the Reactive Oxygen Species 

**Figure 4:**
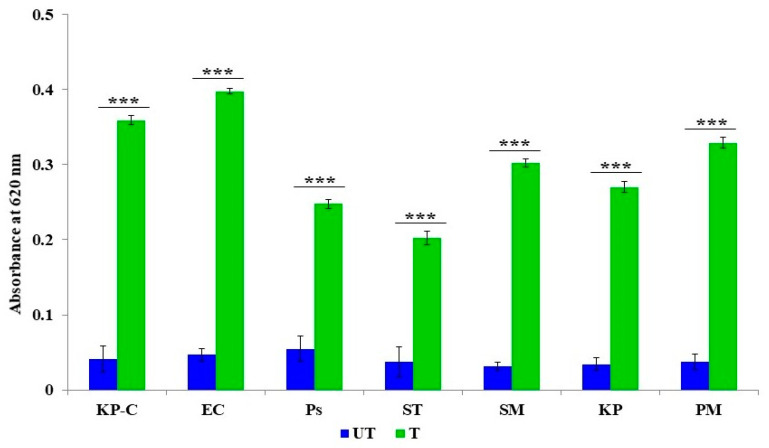
The action of ZnO NPs on the intracellular production of reactive oxygen species. The data are the average of the triplicates and the error bar represents mean ± SD. Compared to ZnO NP free control *** *p* < 0.001.

We examined the ROS produced in ZnO NPs treated and untreated with β-lactam-resistant bacterial strains. All the obtained values were statistically highly significant (*p* < 0.001). The study results demonstrate that intracellular ROS produced in response to ZnO NPs was increased in all tested strains compared to untreated strains (Figure 4). Moreover, for the statistically significant correlation between the treated and untreated strains, the highest ROS production was observed in *S. marcescens*, *P. mirabilis*, *K. pneumoniae E. coli*, and *S. typhi.* The lowest ROS production was observed in *P. aeruginosa.* However, the results confirm that the increased ROS production in treated bacterial cells indicates abnormal cellular metabolisms. 

### 2.5. Membrane Lipid Peroxidation 

**Figure 5:**
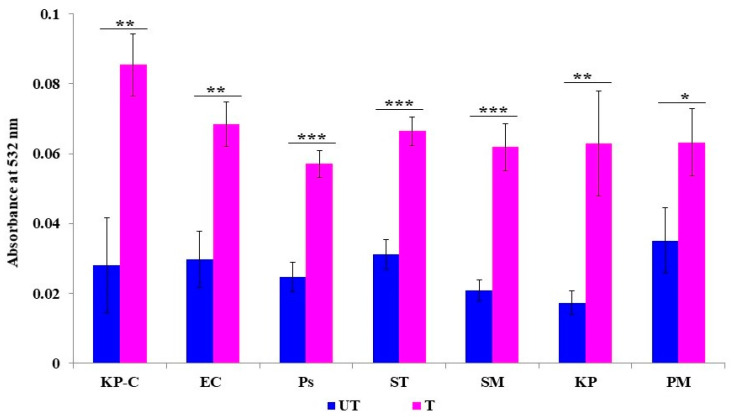
The action of the ZnO NPs on lipid peroxidation. The data are the average of the triplicates and the error bar represents the mean ± SD. Compared to ZnO NP free control * *p* < 0.05, ** *p* < 0.01, *** *p* < 0.001.

The accumulation of ROS leads to bacterial membrane lipid peroxidation, which increases the cell permeability that causes the uncontrolled transport of intra- and extracellular molecules. Lipid peroxidation was assessed by the malondialdehyde quantity, which was the by-product of membrane lipid peroxidation. Figure 5 shows that the level of malondialdehyde was significantly increased in all the ZnO NPs treated with β-lactam-resistant bacterial strains. All the obtained values are statistically significant. The obtained results confirm the effects of the ZnO NPs on the ROS-mediated membrane lipid peroxidation of the tested strains. 

### 2.6. Membrane Damage and Leakage 

**Figure 6:**
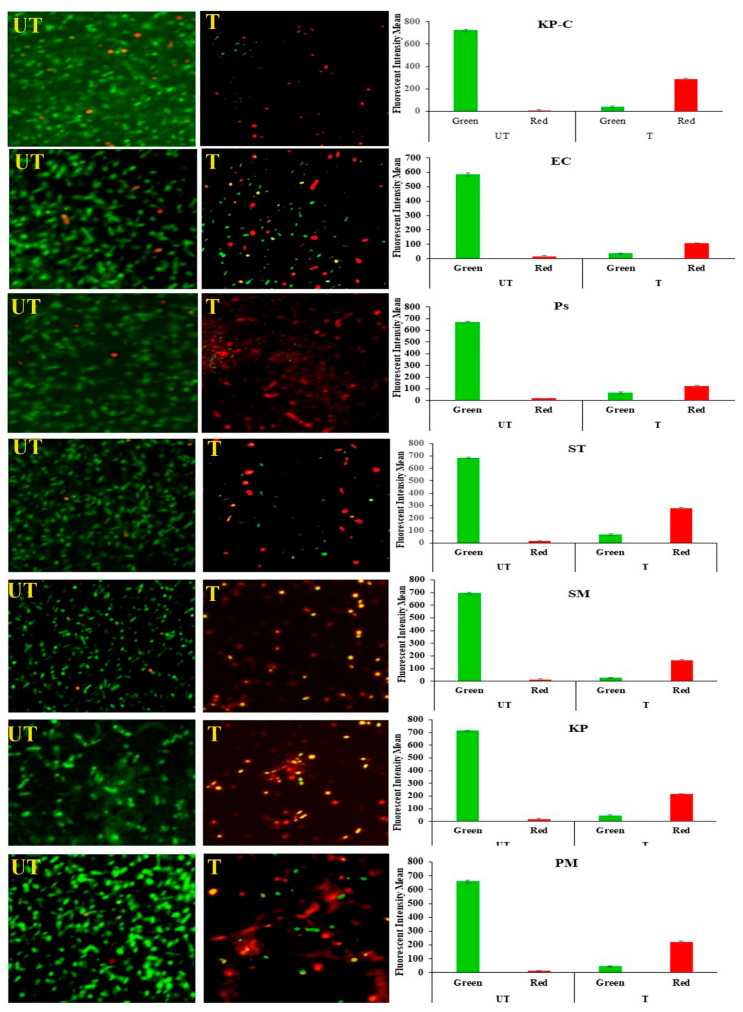
The double staining (SYTO9 and PI) assay for membrane damage; live bacteria with intact membranes appear green, and the injured/damaged bacterial cells appear yellow/red. The bar diagram represents the fluorescent mean intensity.

SYTO9 penetrates all bacterial membranes (intact/injured) and colors the bacterial cells green. PI can only penetrate injured/damaged bacterial cells and color the bacterial cell red. Figure 6 represents the presence of the intact and injured tested bacterial cells. The study results show that all the untreated cells appear green, and the mean fluorescent intensity is high; hence, the experimental setup did not affect the bacterial growth. On the other hand, all the ZnO NPs with treated bacterial cells appear red and the red fluorescent mean intensity is higher than the green fluorescent mean intensity. Hence, the study results confirm that the ZnO NPs cause membrane damage to treated bacterial cells. 

**Figure 7:**
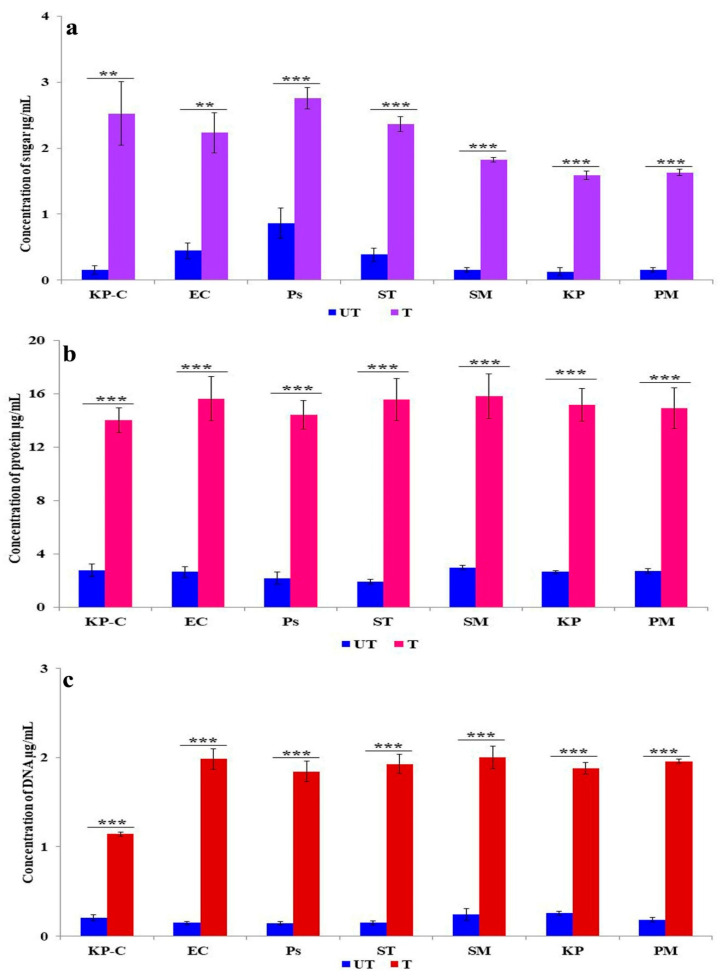
The action of ZnO NPs on beta-lactam-resistant strains in the membrane leakage of (**a**) reduced sugars, (**b**) intracellular proteins, and (**c**) DNA. The data are the average of the triplicates and the error bar represents the mean ± SD. Compared to ZnO NP free control ** *p* < 0.01, *** *p* < 0.001.

The endogenous generation and accumulation of ROS in bacterial cells oxidize the intracellular protein and sugars and cause DNA fragmentation. Figure 7a describes the membrane leakage caused by reducing the sugar in the ZnO NPs with treated and untreated bacteria. Significantly, tenfold increases were observed in *K. pneumoniae*, *S. marcescens*, and *P. mirabilis*; in comparison, lower activity was presented in *S. typhi* (sixfold), *E. coli* (fivefold), and *P. aeruginosa* (threefold) than in the untreated control. Protein leakage is presented in Figure 7b; all the treated β-lactam-resistant bacterial strains expressed a similar pattern of leakage that represented five-to-eight-fold increases than the control. Figure 7c displays the variation of DNA leakage in the treated bacteria. The *E. coli*, *P. aeruginosa*, *S. typhi*, and *P. mirabilis* showed tenfold increases, whereas five-to-eight-fold increases were observed in *K. pneumoniae* and *S. marcescens.* These results suggest that ZnO NPs disrupt the cell membrane integrity and increase cell permeability, hence the acceleration of reducing sugar, protein, and DNA leakage from the cytoplasm in different ways in the different tested strains. 

### 2.7. Transmission Electron Microscopy Analysis 

**Figure 8:**
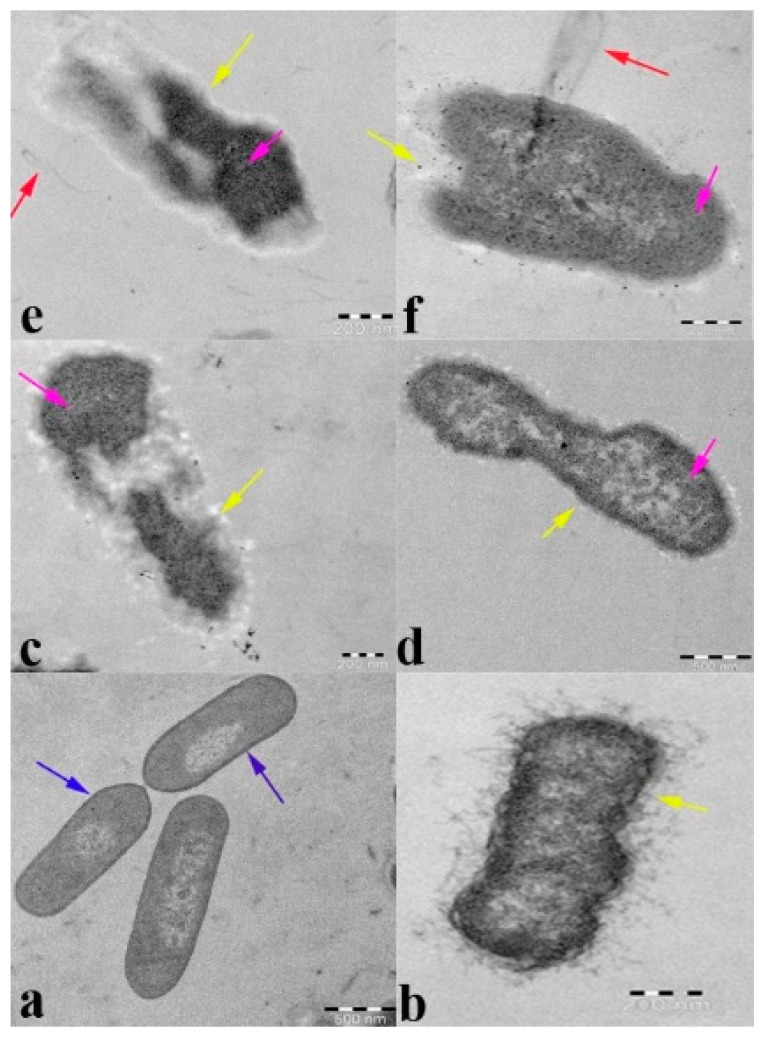
A Transmission Electron Microscopy (TEM) image of beta-lactam-resistant *K. pneumoniae* (**a**). Free control ZnO NPs and (**b**–**e**) treated ZnO NPs. (**b**) Cytoplasmic shrinkage, (**c**) disintegrated cell wall and cell membrane, (**d**) denatured protein appears as a dark electron-dense region, and (**e**,**f**) cytoplasmic leakage. Blue arrow: intact cell wall, yellow arrow: disintegrated cell wall and cell membrane, and violet arrow: denatured protein.

To demonstrate the interaction of ZnO NPs with β-lactam-resistant *K. pneumoniae* cellular events and internal modifications, Figure 8 presents the various cellular events that occur in relation to *K. pneumoniae*, upon being treated with ZnO NPs, compared to the untreated control. Figure 8a shows the untreated β-lactam-resistant *K. pneumoniae* cells that appear as clear and distinct with a uniform morphology. The cytoplasm of the cells that is presented is intact with the semipermeable membrane and cell envelope. Additionally, a thick cell wall with a high lipid bilayer density can be observed, hence ensuring that the experimental conditions of the present study were optimum and cell function was normal without any environmental disturbances. The TEM images of 8b–e represent the various significant modifications that occur in the *K. pneumoniae* cells at 2x MIC of the ZnO NPs. Figure 8b shows that the ZnO NPs cause cytoplasmic shrinkage, appearing as a dense region, lose cellular integrity due to membrane disruption, and cytoplasmic leakage as electron-dense granules surrounding the cell. Figure 8c,d represent the complete disintegration of the cell wall; the cell membrane, cytoplasm, and lipid bilayer appear as translucent, electron-light regions; and the dense aggregation of proteins appear as an electron-dense region in the bacterial cells. Figure 8e shows that the cell consists of a denatured protein that appears as a dark electron-dense region. These results visually confirm the broad-spectrum potential of the ZnO NPs on β-lactam-resistant bacterial strains and provide evidence of β-lactamase inhibition, ROS, the inhibition of membrane lipids, and membrane leakage. 

## 3. Discussion

The hydrolytic potential of β-lactamase is the primary source of bacterial resistance to β-lactam antibiotics. This enzyme can break the β-lactam ring and deactivate β-lactam drugs [33]. The continuous evolution and spreading of β-lactam-resistant bacterial strains have prompted the modern food industry to search for antimicrobial alternatives. Thus, ZnO NPs are commonly considered as potent antimicrobial agents; however, the exact way in which they exert their activity is still speculative [34]. Nevertheless, the present study demonstrates that ZnO NPs can be potent antimicrobials for β-lactam-resistant bacterial strains. The antibacterial test showed that 0.04 mg/mL of ZnO NPs is sufficient to inactivate the *K. pneumoniae* ATCC 700603 strain, which is capable of producing β-lactamase SHV-18. Additionally, similar results were observed for *E. coli*, and *S. marcescens* isolated food pathogenic strains. For the bacterial strains that produce SHV-18 β-lactamase, the relative rate of the hydrolysis/inactivation of cephaloridine was two-fold higher than SHV-7 [35].

SHV-18 differs from SHV-7 by a single amino acid substitution: alanine for serine [36]. However, the hydrolysis/drug inactivation potential was increased in other classes of SHV β-lactamase-producing strains; hence, in our study, foodborne β-lactamase producing *K. pneumoniae*, *P. aeruginosa*, *S. typhi*, and *P. mirabilis* exhibits a higher MBC (0.24 mg/mL) value than the SHV-18 strain (0.2 mg/mL); these variations may occur due to other classes of β-lactamase and other virulence factors. In previous studies on the antibacterial activity of ZnO NPs against non-resistant Gram-negative bacterial strains, it was observed that the inhibition dose was 0.015 mg/mL against *E. coli* and *S. typhimurium*, and, in the case of *K. pneumoniae*, the lowest dose of 0.005 mg/mL was sufficient to inactivate these strains [37,38]. However, in the present study, the growth was strongly inhibited at the concentration range of 0.04 to 0.08 mg/mL, against tested β-lactam-resistant bacterial strains. The study results demonstrate that ZnO NPs can be effective antimicrobial agents in the case of β-lactam-resistant bacterial food pathogens. Nonetheless, their success is strongly associated with the class of β-lactamase they produce and dependent strains, since the differences in terms of MIC and MBC can be observed among the tested strains. Additionally, many other factors strictly related to each strain, as demonstrated in the case of *S. typhi* and *P. mirabilis* that were equipped with an efflux pump, altered the cell membrane, fimbriae, an array of enzymes, and reduced compounds for detoxification; thus, increased concentrations of MIC and MBC were observed in our test results [39,40]. It has been reported that Salmonella is capable of surviving and proliferating in diverse niches [41], due to its protective response, which is known as its antigenic property; thus, it has numerous serotypes [42]. *P. aeruginosa* virulence factors of the quorum-sensing protein, elastase, and pigment production facilitate the bactericidal action of ZnO NPs at a slightly increased concentration [43]. Nanoparticles with different sizes consist of a different surface area-to-volume ratio; the physical interactions between nanoparticles and bacterial cells were facilitated [44], which featured the bactericidal action of ZnO NPs and the production of reactive oxygen species (ROS) that play an important role in biological applications [45]. The different sizes of the nanoparticles may act differently on bacterial cells and cumulatively cause cell death; however, smaller-sized nanoparticles exhibit a greater effect [46]. 

The bacterial cell membrane is the first barrier against ROS attachment; however, the normal metabolism of ROS is regulated via a cellular antioxidant defense system to maintain equilibrium in the redox system. Thus, the ROS levels were low in all the untreated (Figure 4) bacterial strains [47]. The enhanced production of ROS in the ZnO NPs treated cells caused the excess accumulation of ROS, and eventually diminished the cellular GSH pool, leading to inadequate antioxidants and an over-accumulation of ROS, resulting in abnormal metabolism. We investigated lipid peroxidation, membrane damage and leakage, and the β-lactamase inhibition assay to obtain a comprehensive understanding of the accumulated ROS. Our results show that the exposure cells of the ZnO NPs produce a high level of malondialdehyde (Figure 5), which is a highly reactive by-product of membrane lipid peroxidation, which likely contributes to the disintegration of the bacterial cell membrane (Figure 8c) [48]. This, together with the physical interaction between the ZnO NPs and bacterial cells, leads to cell membrane dysfunction, as evidenced by the damage to the membrane (Figure 6), and cytoplasm leakage caused by reduced sugars, proteins, and DNA (Figure 7a–c). This phenomenon allows the ZnO NPs to enter the cytosol to interact with cytoplasmic proteins and enzymes, leading to further ROS generation, and causing internal cellular protein aggregation and enzyme inhibition [49]. Figure 3 presents the evidence of β-lactamase activity inhibition by the ZnO NPs. The β-lactamase protein is involved in the mechanisms for many Gram-negative bacteria against a wide range of β-lactam antibiotics [50]. It has been reported that the ionic metal properties of the ZnO NPs inhibit the enzyme activity of bacteria, and zinc plays a catalytic function and structural role in a large number of macromolecules and enzymes. In turn, structures called zinc fingers provide a unique scaffold that allows protein subdomains to interact with DNA and proteins [51,52]. Different β-lactam bacterial strains produce a different class of these enzymes. Each has a different catalytic efficiency, so that the rates for β-lactam antibiotics can approach the limit of diffusion control [53]. Hence, a variation was observed in the β-lactamase inhibition assay between the *K. pneumoniae* isolate and the ATCC strain; likewise, the inhibition rate was different among the other isolates. However, our study results found that all the treated ZnO NP strains that were tested, exhibited a β-lactamase inhibition. The mechanisms underlying the ZnO NPs act as nano inhibitors that enhance the ROS-mediated surface functional modification and significantly alter β-lactamase activity through multivalent interactions or steric hindrances [54]. More research is needed to clarify these phenomena in the future. 

By examining ROS production, membrane lipid peroxidation, membrane leakage, β-lactamase inhibition, cell morphology, and membrane integrity, we found that all aspects were affected by the ZnO NPs and demonstrated a broad-spectrum antibacterial activity. Dramatic changes in the morphology of *K. pneumoniae* were revealed by the TEM micrograph (Figure 8). The primary antimicrobial response of the ZnO NPs begins with the release of Zn2^+^ ions, their ability to penetrate the cell, and their intracellular response and enhanced ROS production [29]. The consequences of the concentration gradient of ROS caused the inactivation of proteins/enzymes, followed by a denatured protein aggregation that appeared in the TEM micrograph as a dark electron-dense region (Figure 8e). The lipid peroxidation (Figure 5) of polyunsaturated fatty acids, such as cell membrane phospholipids, leads to membrane disruption (Figure 6 and Figure 8f) and disintegration (Figure 8c) [55]. Further mediated oxidative stress may cause DNA and cytoplasmic leakage, including reduced sugars, DNA, and proteins (Figure 7) [54]. Previous studies suggested that a low concentration of ZnO NPs cannot have a negative impact on the digestive system of human beings. The consumption of zinc via food can protect the stomach and intestinal tract from damage by *E. coli* [56]. However, Zn2+ ions support the activation of human digestive enzymes, such as carboxypeptidase, carbonic anhydrase, and alcohol dehydrogenase [57]. In the present study, the synthesized ZnO NPs have a spherical shape with varying size exert a high dispersion of antimicrobial activity [51]; hence, the synthesized ZnO NPs possess potent antibacterial activity against β-lactam-resistant Gram-negative food pathogens. Additionally, the adopted method for the synthesized ZnO NPs, such as pH and temperature, is suitable for antimicrobial-based food packing applications to reduce the risk of pathogen contaminations [58]. 

## 4. Materials and Methods

### 4.1. The Synthesis of the ZnO NPs and Their Characterization

The ZnO NPs were prepared using zinc acetate dihydrate as a precursor. Approximately 100 mL of 0.01 M zinc acetate aqueous solution was prepared, and its pH was adjusted to 10 using NH_3_OH. The solution was treated in a microwave oven at 900 W for 15 min. Consequently, the obtained white color precipitates were washed with water twice by centrifugation. Subsequently, a white pellet was dried at 70 °C. The synthesized material was subjected to characterization. The crystalline nature of the synthesized ZnO NPs was studied using X-ray diffraction (Shimadzu XRD 6000) with Cu Kα radiation (λ = 1.5406 Å). The optical behavior of the ZnO NPs was analyzed using UV-Vis-NIR spectroscopy (Carry 5000, Agilent Technologies, Santa Clara, CA, USA). The sample surface morphological features were observed using a scanning electron microscope (Jeol JSM 6390). The particle-size distribution was analyzed using a dynamic light scattering zeta size analyzer (Zetasizer Nano-ZS90, Malvern, UK).

### 4.2. Bacterial Strains 

The bacterial strains were isolated from spoiled meat and chicken samples collected from a local market in the Batha region of Riyadh, Saudi Arabia. Each sample was inoculated on two selective media: MacConkey broth and a subculture on MacConkey agar that contained ceftazidime (1 mg/L) and cefotaxime (1 mg/L). The isolated pathogens, such as *Escherichia coli*, *Pseudomonas aeruginosa*, *Salmonella typhi*, *Serratia marcescens*, *Klebsiella pneumoniae*, and *Proteus mirabilis*, were identified by 16s rRNA sequencing, and β-lactam drug resistance and ESBL production were evaluated using Clinical and Laboratory Standards Institute (CLSI) guidelines [59]. The *E. coli* ATCC 25922 was used (non-ESBL producer) as a negative control, and *K. pneumoniae* ATCC 700603 was used as an ESBL-producing control strain in the present study. All the strains were maintained in nutrient agar slants at 4 °C. 

### 4.3. The Minimal Inhibitory Concentration (MIC) and Minimal Bactericidal Concentration (MBC) Assay 

The anti-microbial activity of the ZnO NPs was tested by determining the minimal inhibitory concentration (MIC) and the minimal bactericidal concentration (MBC), according to the standard microdilution method (CLSI), with some modifications [60]. Briefly, the bacteria were grown in Mueller–Hinton (MH) broth (Hi-media, Mumbai, India), until the mid-log growth phase, and the initial suspension was adjusted to reach a final density of 3 × 10^5^ CFU/mL. A volume of 10µL of different strains of diluted bacterial suspensions was added to respective wells in a 96-well plate that contained a different concentration of ZnO NPs ranging from 0.02 to 0.4 mg/mL. The inoculated plates were incubated at 37 °C for 24 h. the MIC was defined as the lowest concentration of the ZnO NPs that inhibits the visible growth. The MBC was determined by plating 10 µL of the samples from wells on MH agar, and the plates were incubated at 37 °C for 24 h. After incubation, no growth on the MH agar was considered as an MBC. Imipenem (IMP) was used as a positive control. 

### 4.4. The Inhibition of β-Lactamase Production 

Initially, β-lactamase inhibition activity was induced by treating bacterial culture with imipenem (0.08 mg/L) for 6 h [61]. Subsequently, the cells were washed twice with fresh MH broth by centrifugation at 5000× *g* for 10 min and diluted into MH broth to finally attain OD_600 nm_ 0.07 (1 × 10^7^ CFU/mL confirmed upon a retrospective plate count on the MH agar). The bacterial suspension was treated with the ZnO NPs at an MIC concentration in the MH broth at 37 °C for 18 h. The untreated bacterial suspension was used as a control. After the incubation, bacterial pellets were obtained by centrifugation at 10,000× *g* for 30 min. The collected pellet of each bacterium was suspended with 1 mL of cell lysis buffer per mg sample. Subsequently, the samples were sonicated for 5 min. The samples were immersed in ice during the sonication process and centrifuged at 10,000× *g*, at 4 °C for 20 min. The β-lactamase activity was indicated by measuring the absorbance at 490 nm for 15 min after adding the collected 100 μL of the obtained β-lactamase samples to the respective tubes that contained nitrocefin (50 μM) in 1 mL of 0.1 M phosphate buffer. The β-lactamase activity level was compared to the untreated control. 

### 4.5. The Reactive Oxygen Species (ROS) Assay 

The reactive oxygen species produced by the treated and untreated β-lactamase-resistant bacterial cells were estimated by the Nitro Blue Tetrazolium (NBT) assay, as previously described [62,63]. Briefly, the bacterial suspension was treated with the ZnO NPs (2x MIC/mL^−1^); both the treated and untreated (control) bacterial suspensions were incubated in an orbital shaker at 80 rpm and 37 °C for 6 h. Following the incubation, the bacterial pellet was collected by centrifuging at 10,000× *g* for 15 min. The collected pellet was mixed with a 2% NBT solution and incubated in dark conditions at 37 °C for 1 h. Subsequently, the incubation pellet was collected by centrifuging at 5000× *g* for 5 min and the extracellular NBT was removed by washing with PBS, followed by methanol. The NBT that was deposited inside the cells was obtained via solubilizing the cell membrane with a 2 M potassium hydroxide solution. To dissolve the formazan crystals, 50% of a dimethylsulfoxide (DMSO) solution was added and incubated at 37 °C for 15 min, and then centrifuged at 5000 g for 5 min. The collected supernatant was transferred to 96 micro-well plates, and the absorbance was measured at 620 nm using a microplate reader. 

### 4.6. The Membrane Lipid Peroxidation Assay 

Lipid peroxidation can be measured by the thiobarbituric acid reactive substance test (TBARS) [64]. The oxidative stress causes the formation of unstable lipid peroxide in β-lactam-resistant bacterial cells that decompose to form reactive compounds, such as malondialdehyde (MDA). Briefly, the bacterial suspension was treated with the ZnO NPs (2x MIC/mL^−1^) and incubated in an orbital shaker at 80 rpm and 37 °C for 6 h. Following the incubation, the bacterial suspension was centrifuged at 10,000× *g* for 30 min; the collected pellet was washed and resuspended in 10% of SDS, then 20% of acetic acid was added and incubated at 37 °C for 10 min. Following incubation, the thiobarbituric acid (TBA) buffer (2 M NaOH and 0.8% TBA solutions) was added and incubated at 95 °C for 60 min. After cooling to 25 °C, the reaction mixture was centrifuged at 5000× *g* for 15 min. The absorbance of the supernatant was recorded at 532 nm using a microplate reader. 

### 4.7. The Membrane Damage and Membrane Leakage Assay 

The bacterial membrane damage caused by the ZnO NPs was assessed by using the LIVE/DEAD BacLight kit (Invitrogen, Waltham, U.S.A). This kit consists of the membrane-permeable stain SYTO9 and impermeable stain, propidium iodide (PI), which enter the cell through a permeabilized/damaged membrane. The green and red fluorescent intensity of the double-stained treated and untreated ZnO NP cells was measured using a confocal laser scanning microscope (ZEISS, Oberkochen, Germany). 

The membrane leakage, such as the reduced sugars, proteins, and DNA, in the intracellular cytosol were analyzed for both the treated and untreated β-lactam-resistant bacteria. Briefly, bacterial suspension was treated with the ZnO NPs (2x MIC/mL^−1^) and incubated in an orbital shaker at 100 rpm, at 37 °C for 6 h. Following incubation, the bacterial suspension was centrifuged at 10,000× *g* for 30 min at 4 °C, and the supernatant was collected and stored at −20 °C. The dinitrosalicylic acid test was employed to quantify the reducing sugar, the protein was estimated by the Bradford method, and the DNA was estimated using the absorption spectra at 260 nm, as described in the previously published protocol [63]. 

### 4.8. Transmission Electron Microscopy (TEM) Analysis 

Both the treated and untreated ZnO NP (control) β-lactam-resistant bacterial cells were centrifuged at 2000 rpm for 10 min. The collected pellets were placed in a sterile Eppendorf tube that contained 0.1 M of sucrose with buffered 2.5% glutaraldehyde. Then, the post-fixation was performed for each sample using 1% of osmium tetroxide and incubated for 12 h. The fixative and the buffer were removed by centrifugation; then the cells were rinsed with MilliQ water, and 1% of uranyl acetate was used for staining. The fixed specimen samples were dehydrated using 20%, 40%, 60%, 90%, and 100% of ethanol series and then propylene oxide was added for 20 min. The dried cell blocks were infiltrated by a mixture of 1:1 (*v*/*v*) propylene oxide and eponate 12 resin for 1 h at 37 °C, then by a mixture of 1:2 (*v*/*v*) polypropylene/resin overnight at room temperature on a rotator. Finally, the cells were infiltrated in 2 changes of 100% eponate 12 resin over 2 to 6 h at 37 °C [65]. Following the infiltration, plastic capsules were used to embed the tissue blocks, and then polymerized for 12 h at 60 °C. Ultra-thin sections (70 nm) were prepared using an ultramicrotome (Leica EM UC6, Leica Microsystem GmbH, Vienna, Austria). The cells were stained in 2% of aqueous uranyl acetate for 20 min, washed with distilled water, stained in Reynold’s lead citrate for 15 min, and washed again with distilled water. After air drying, TEM images of the cells were obtained using a JEOL transmission electron microscope.

### 4.9. Statistical Analysis 

All the experiments were performed in triplicates. The obtained values are expressed as the mean ± SD. Statistical significance was calculated using one way ANOVA with Microsoft Excel 2010, and the value of *p* < 0.05 is considered as statistically significant. 

## 5. Conclusions 

Ultimately, it can be concluded from these results that the bactericidal activity of the ZnO NPs against β-lactam-resistant Gram-negative food pathogens is mediated by Zn2+ ion-induced oxidative stress, mechanisms via lipid peroxidation, and membrane damage, subsequently resulting in depletion, which leads to β-lactamase enzyme inhibition, intracellular protein inactivation, DNA damage, and eventually cell death. Based on the findings of the present study, ZnO NPs can be recommended as potent antibacterial agents against β-lactam-resistant Gram-negative pathogenic strains for food production and processing. However, the biosafety, long time exposure, and toxicity of ZnO NPs at an accumulated concentration need to be explored. Thus, as a future perspective, it is important that further research must be conducted in this regard. 

## Figures and Tables

**Table 1:** The minimal inhibitory concentration (MIC) and minimal bactericidal concentration (MBC) of ZnO NPs against β-lactam-resistant bacterial food pathogens.

Bacterial Strains	MIC (mg/mL)	MBC (mg/mL)
*E. coli* ATCC 25922	0.04	0.12
*K. pneumoniae* ATCC 700603	0.04	0.2
*E. coli*	0.04	0.2
*P. aeruginosa*	0.04	0.24
*S. typhi*	0.08	0.24
*S. marcescens*	0.04	0.2
*K. pneumoniae*	0.04	0.24
*P. mirabilis*	0.08	0.24

## Data Availability

The data presented in this study are available on reasonable request from the corresponding authors.

## Sample Availability

Samples of the compounds are available from the authors.
